# Meningococcal factor H-binding protein: implications for disease susceptibility, virulence, and vaccines

**DOI:** 10.1016/j.tim.2023.02.011

**Published:** 2023-08

**Authors:** Wearn-Xin Yee, Grace Barnes, Hayley Lavender, Christoph M. Tang

**Affiliations:** 1 Sir William Dunn School of Pathology, University of Oxford, South Parks Road, Oxford OX1 3RE, UK

**Keywords:** *Neisseria meningitidis*, complement factor H, CFHR, fHbp, vaccines, GWAS

## Abstract

*Neisseria meningitidis* is a human-adapted pathogen that causes meningitis and sepsis worldwide. *N. meningitidis* factor H-binding protein (fHbp) provides a mechanism for immune evasion by binding human complement factor H (CFH) to protect it from complement-mediated killing. Here, we discuss features of fHbp which enable it to engage human CFH (hCFH), and the regulation of fHbp expression. Studies of host susceptibility and bacterial genome-wide association studies (GWAS) highlight the importance of the interaction between fHbp and CFH and other complement factors, such as CFHR3, on the development of invasive meningococcal disease (IMD). Understanding the basis of fHbp:CFH interactions has also informed the design of next-generation vaccines as fHbp is a protective antigen. Structure-informed refinement of fHbp vaccines will help to combat the threat posed by the meningococcus, and accelerate the elimination of IMD.

## Highlights

*Neisseria meningitidis* is a common human commensal which occasionally causes invasive meningococcal disease (IMD). The bacterium recruits the negative complement regulator complement factor H (CFH) to its surface by expressing factor H-binding protein (fHbp); this protects the meningococcus from the human complement system.
fHbp is regulated *in vivo* by environmental cues (such as oxygen levels and temperature), and interactions between fHbp and CFH and other host complement proteins (encoded by the *cfh* locus) are central to host susceptibility to IMD.
A recent bacterial genome-wide association study highlighted the role of fHbp determining whether strains harmlessly colonize an individual or cause IMD.
Knowledge of fHbp structure:function is being exploited to develop next-generation vaccines against the meningococcus, and to understand why infection can result in harmless colonization or IMD in certain individuals.

## Introduction

fHbp is a virulence factor expressed by the human-specific pathogen *N*. *meningitidis*, a leading cause of meningitis and sepsis worldwide which frequently asymptomatically colonizes the human upper airway. fHbp binds human CFH, a negative regulator of the complement system, and has multiple roles during meningococcal infection. fHbp was initially identified as a vaccine antigen, named GNA1870 [1] or LP2086 [2]. It was then shown that the meningococcus binds CFH to its surface via an ~33 kDa protein, promoting bacterial survival in serum [3]. Subsequently, the protein recruiting CFH was identified as GNA1870/LP2086 [4], and renamed fHbp. Here, we describe the features of fHbp that enable it to recruit hCFH. We highlight the consequences of this intimate interaction on the relationship between the meningococcus and its human host, and how disease occurs infrequently in some individuals, while others are harmlessly colonized. Furthermore, we discuss the consequences of fHbp:CFH binding on the predilection of *N. meningitidis* isolates to cause IMD. The modification of fHbp in next-generation vaccines is discussed to increase vaccine coverage in circulating strains.

## fHbp and CFH: structure and function

fHbp is expressed on the bacterial surface where it influences host:pathogen interactions. Initially, fHbp is translated as a pro-protein which is cleaved, lipidated [1,2], then inserted into the external leaflet of the outer membrane through the action of Slam, an outer membrane translocon [5]. fHbp is composed of two β-barrels [6], tethered to the bacterial surface via its lipid modification. Each β-barrel consists of eight anti-parallel β-strands joined by a short linker [6]. The C-terminal β-barrel of fHbp is stable and melts at temperatures above 80°C, while the N-terminal barrel adopts a more open conformation and melts at significantly lower temperatures [7].

fHbp binds hCFH with nanomolar affinity [4,6]. CFH regulates the complement system and allows appropriate responses against invading microbes while ensuring that host cells are protected from complement-mediated attack [8]. The effect of CFH on complement results from its role in the complement alternative pathway (AP), in which it regulates the key complement component, C3b [9]. C3b is generated by C3 convertases, and covalently binds to microbial surfaces [8]. Bound C3b is an opsonin promoting phagocytosis, and initiates the complement terminal pathway (TP) leading to cell lysis [10,11]. CFH promotes C3b cleavage by factor I and interferes with the AP C3 convertase [8]. Therefore, recruitment of CFH protects the meningococcus from complement-mediated phagocytosis and lysis [3,4,12].

CFH is abundant in serum and present at mucosal surfaces [13], where it can bind fHbp on the meningococcus. CFH is a modular protein consisting of 20 repeating complement control protein modules (CCPs) [8]; each CCP has approximately 60 amino acids [8] and is joined to neighboring CCPs by three to eight amino acids [8]. Different CCPs of CFH have distinct functions. CCPs 1–4 bind C3b, enhancing factor I cleavage of C3b [8]. In addition, these CCPs accelerate the decay of C3bBb, the C3 convertase of the AP [14]. CCPs 6–7, and 19, 20, are instead involved in recognizing host cell-surface molecules, including glycosaminoglycans (GAGs) which are present on human endothelial cells [15,16].

**Figure 1:**
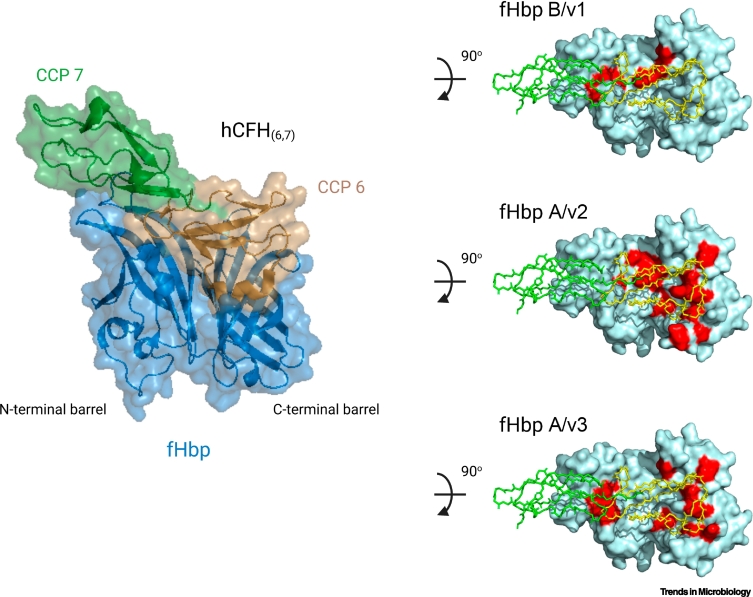
Structure of factor H-binding protein (fHbp) and residues required for high-affinity interactions with human complement factor H (CFH). The image on the left shows fHbp (blue) binding to CFH complement control proteins (CCPs) 6 and 7 (gold and green, respectively). Images on the right show alanine substitution of amino acids in red of family B/variant 1 (B/v1), family A/variant 2 (A/v2), and family A/variant 3 (A/v3) that cause a fivefold or greater decrease in binding to CFH CCPs 6 and 7 (yellow and green, respectively) modeled on the structure of fHbp A/v3 PDB: 4AYI (cyan).

fHbp interacts with CFH via CCPs 6 and 7 [6] which allows CFH recruitment without impairing its ability to downregulate complement. The structure of fHbp with CFH CCP 6 and 7 revealed extensive interactions between the two molecules that span a large area [6]. In an elegant example of ligand mimicry, charged amino acids in fHbp bind at precisely the same site in CFH as charged saccharides of host GAGs [6]. This sets up potential competition between pathogen and host molecules for CFH. Of note, the affinity of CFH for fHbp is far higher than for host GAGs [6]. Therefore, the meningococcus could sequester CFH from cells of the vascular endothelium, rendering them sensitive to complement lysis, and potentially exacerbating vasculitic lesions seen in IMD [17]. Substitution of single amino acids of fHbp or CFH at their interface can lead to a marked reduction in binding (Figure 1) [6,7]. The high-affinity interaction between CFH and fHbp at the bacterial surface allows *N. meningitidis* to regulate host complement activation and facilitate its survival within human serum, and cause disease.

## Regulation of *fhbp*

**Figure 2:**
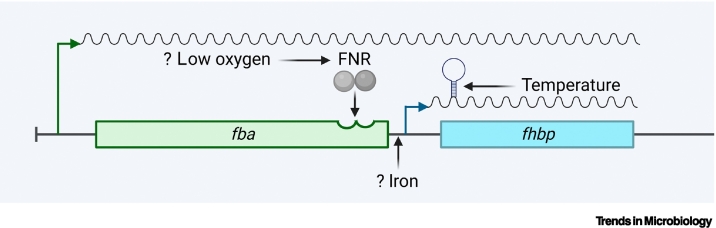
Regulation of factor H-binding protein (*fhbp*) expression. *fhbp* is transcribed as a mono- or bi-cistronic mRNA with promotors upstream of *fhbp* or *fba* (encoding fructose-1,6-bisphosphate aldolase), respectively. A putative fumarate and nitrate reductase (FNR) box has been identified in the –35 sequence of the *fhbp* promoter, and overexpression of FNR increases the levels of *fhbp*. Surface fHbp also rises in response to increasing temperature, mediated by an RNA thermosensor in the *fhbp* mRNA. Iron also plays a role in the transcriptional regulation of *fhbp* with a putative Ferric Uptake Regulator (Fur) box identified in the promotor.

fHbp expression responds to environmental cues that are found in the human host (Figure 2). The *fhbp* gene is located 157 bp downstream of *fba*, which encodes fructose-bisphosphate aldolase [1], and is transcribed as a mono- and bi-cistronic mRNA [18]. The ribosomal binding site (RBS) of fHbp mRNA is 45 nucleotides upstream of the translational start codon; putative –10 and –35 consensus sequences are consistent with regulation by the σ^70^ family of sigma factors [18].

There is evidence that oxygen availability might affect fHbp expression. The transcriptional regulator fumarate and nitrate reductase (FNR) orchestrates responses of bacteria to low oxygen; FNR dimerizes under anaerobic conditions, allowing binding to DNA sequences (FNR boxes), modulating expression of downstream genes [19]. A putative FNR box was found overlapping the –35 sequence of *fhbp*. While overexpression of constitutively active FNR in the meningococcus upregulates fHbp expression, there was no major change in either *fhbp* mRNA or protein levels when bacteria were grown anaerobically for 30 min, so the physiological relevance of FNR binding remains uncertain [18].

Iron levels also influence fHbp expression, although this is strain-dependent. Iron is essential for bacterial metabolism, but is in limited supply at mucosal surfaces [20]. In iron-depleted conditions, most meningococcal strains have reduced levels of *fhbp* mRNA, although transcription in isolates belonging to the cc32 lineage increases [21]. The effect of iron was particularly marked in strains containing a 181 bp insertion element downstream of the –10 sequence [21].

fHbp expression is also affected by temperature (Figure 2) [22]. The meningococcus is subjected to temperature gradients in the upper airway, and to higher temperatures during IMD. The response of fHbp to temperature is mediated by an RNA thermosensor [22]. At lower temperatures, two anti-ribosome binding sites base-pair with the RBS in *fhbp* mRNA, forming a hairpin structure, limiting translation by preventing ribosome access to the mRNA. At higher temperatures, the heat labile hairpin melts, allowing translation and increased fHbp levels. Therefore, sequences in the *fhbp* mRNA act as a temperature-dependent molecular rheostat [23], indicating that less fHbp will be on the surface of bacteria in the upper airway compared to when they are in the warmer environment of the bloodstream [24].

*N. meningitidis* strains vary in the amount of fHbp on their surface, with up to 15-fold differences seen [25]. Additionally, rare disease isolates carry a frameshift mutation in *fhbp* abolishing fHbp expression [26]. Based on differences in their amino acid sequence, fHbps can be assigned to two families (A or B) or three variant groups (v1, 2, or 3) [1,2]. Generally, B/v1 fHbps are expressed at higher levels compared with A/v2 or v3 fHbps; this has been attributed to differences in the *fba–fhbp* intergenic region, affecting the mono-cistronic promoter [25], so typing systems based on this sequence have been proposed to predict surface levels of fHbp [25,27]. These schemes might have implications for bacterial virulence and their coverage by fHbp-based vaccines.

## Human specificity of fHbp

fHbp specifically binds to hCFH, and not CFH from mice or most primates. This feature potentially contributes to the exquisite adaptation of the meningococcus to its human host. Amongst different primates, fHbp barely binds to CFH from rhesus macaques despite its similarity with hCFH [28]. fHbp also does not bind to murine CFH (mCFH) at physiologically relevant concentrations [7] making rodent models challenging. Interestingly, modifying mCFH CCPs 6 and 7 with 13 amino acids found in hCFH failed to confer high-affinity fHbp binding, as [7] the orientation of CCPs 6 and 7 is distinct in mCFH and hCFH [7], explaining why extensive replacement of mCFH residues did not enable fHbp binding.

Therefore, caution must be exercised when interpreting results from transgenic mice with added hCFH. CFH interacts with many complement components (e.g*.*, factor I, C3, and C3b [8]), while CCPs 6 and 7, and 19 and 20, recognise polymorphic host glycans [8,16]. Therefore, hCFH might not act appropriately in a heterologous immune and vascular system. Furthermore, the expression of additional hCFH (without removing endogenous mCFH) can lead to supraphysiological CFH levels, and excessive regulation. As an alternative, transgenic mice have been generated lacking their endogenous CFH locus but expressing mCFH with human CCPs 6–8 (allowing interaction with fHbp); as the remainder of CFH is of murine origin, the mouse:human chimeric CFH acts in a physiologically relevant manner [7].

## fHbp, CFHRs, and host susceptibility

The importance of the complement system in protection against IMD is manifest from the dramatically increased risk of IMD among rare individuals and families with inherited complement deficiencies [29]. This is further highlighted by the susceptibility to IMD of people receiving anti-C5 blocking monoclonal antibodies (mAbs) for the treatment of paroxysmal nocturnal hemoglobinuria [30], demonstrating the importance of the TP in protection against invasive disease [17]. However, these conditions do not explain the majority of cases of fulminant invasive disease which occur in individuals without an obvious complement defect [31].

**Figure 3:**
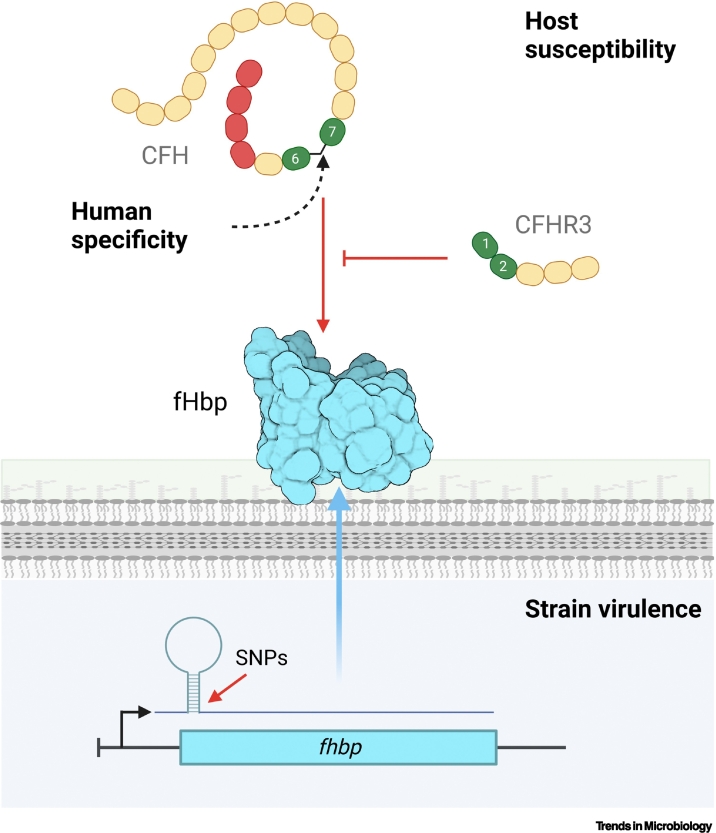
The role of factor H-binding protein **(**fHbp), human complement factor H (CFH), and CFH-related protein 3 (CFHR3) and the development of invasive meningococcal disease (IMD) versus carriage. Host genome-wide association studies (GWAS) identified polymorphisms in CFH and CFHR3 that affect susceptibility to IMD. CFHR3 competes with CFH for binding to fHbp but lacks complement regulatory activity. Bacterial single-nucleotide polymorphisms (SNPs) associated with isolates causing IMD versus carriage are located near the ribosome-binding site (RBS) of *fhbp* mRNA. The orientation of complement control proteins (CCPs) in CFH enables binding of human but not murine CFH by fHbp.

A GWAS identified a region encoding CFH and CFH-related proteins (CFHRs) associated with IMD (Figure 3) [32]. There are five CFHRs (CFHR1–5) encoded by the CFH locus. Analysis of patients and controls identified single-nucleotide polymorphisms (SNPs) in CFH and CFHR3 significantly associated with IMD [32]. One SNP in CFH, rs1065489, encodes a nonsynonymous substitution, and the CFHR3 polymorphism (rs426736) lowers the risk of IMD. Subsequent studies confirmed these associations, and identified a further SNP (rs193053835) in CFH [33,34].

The mechanism by which CFH SNPs influence the risk of IMD has not yet been fully determined; the common CFH Y402H polymorphism in CCP 7, which is involved in age-related macular degeneration [35], is adjacent to the fHbp binding site but does not affect CFH:fHbp interactions [6]. By contrast, the role of CFHR3 has been investigated. CFHRs are composed of fewer CCPs than CFH, and lack regions related to CCPs 1–4 of CFH associated with functional activity; therefore, the CFHRs lack decay-accelerating and cofactor activity [36]. Instead, CFHRs can act as antagonists of CFH, by competing with CFH for binding to surface receptors without reducing C3b activation/deposition [12,37]. Therefore, CFHRs are likely to fine-tune complement activation by modulating CFH activity.

CFHR3 binds *N. meningitidis* fHbp via CCPs 1 and 2 [12]. As a result, CFHR3 acts as a competitive antagonist of CFH for binding to fHbp (Figure 3). Interestingly, B/v1 fHbp preferentially binds CFH over CFHR3, likely because of differences in the way fHbp variants engage CFH (Figure 1). This is functionally significant, as the survival of isogenic strains expressing B/v1 fHbp in serum is increased compared with strains expressing other variants. This might explain the prevalence of B/v1 fHbp expressing strains among disease isolates [38]. Furthermore, a protective CFHR3 SNP, rs75703017 was associated with lower CFH concentrations; evidence indicates that sequences around CFHR3 include a regulatory region that affects CFH transcription [39]. Of note, CFHR3 can be lost along with CFHR1 [40], with this deletion being common in individuals from Northern Nigeria [41], where epidemics of meningococcal disease can start [42]. In the future, it will be interesting to see whether this copy number variation (CNV) affects the risk of IMD in sub-Saharan countries with high rates of disease [43].

## Bacterial GWAS: fHbp sequence to RNA structure and function

IMD is one of many infections caused by a bacterium which usually harmlessly infects individuals, raising the issue of what specific features disease-causing bacteria possess compared with carriage isolates. This was addressed by a recent bacterial GWAS. There is a wealth of whole-genome sequences (WGSs) for *N. meningitidis* in part due to the Meningitis Research Foundation-Genome Library (MRF-GL) which contains only disease isolates [44], and large carriage studies which recover isolates causing harmless colonization [45,46]. Analysis of WGS highlights the population structure of *N. meningitidis*, with strains belonging to certain lineages (e.g*.*, cc32, cc41/44, cc11, cc269) responsible for disease. By contrast, more diverse isolates colonize healthy individuals [44,45]. Therefore, the population structure of *N. meningitidis* must be considered to derive causal links between bacterial polymorphisms and IMD/carriage [31].

For GWAS, an initial discovery study was performed on cc11 IMD and carriage isolates, then replicated in a 1046 IMD and 249 carriage isolates belonging to cc41/44 [47]. Results demonstrate that meningococcal virulence is polygenic, with hits found affecting several known virulence factors including the capsule and TspA. The most significant hits were located in the *fba–fhbp* region. SNPs mapped to near the RBS of *fhbp* mRNA; importantly, two nucleotide changes altered the expression and thermoregulation of fHbp expression [47]. Therefore, bacterial and human GWAS of IMD mirror each other, revealing that the interface between fHbp and hCFH/CFHR3 determines strain virulence and host susceptibility.

Analysis of isogenic strains also demonstrates that fHbp levels are influenced by other polymorphisms in the *fba–fhbp* intergenic region [48]. In some strains, changes in the promoter of certain strains abrogate thermosensing, potentially by strengthening the secondary mRNA structures at the RBS. Furthermore, a correlation was found between high predicted fHbp expression in meningococcal isolates from cases (*n* = 2139) compared with controls (*n* = 2977) [48]. However, the data were not stratified for the population structure, making it difficult to draw casual links.

## Refinement of fHbp-based vaccines

Conjugate protein:polysaccharide vaccines have been a major success in preventing disease caused by *N. meningitidis* expressing serogroup A, C, W and Y; a pentavalent vaccine containing a serogroup X conjugated polysaccaride is also in clinical development [49]. However, other approaches are required for serogroup B strains, which cause most endemic disease in wealthy countries; the serogroup B capsule is a mimic of a host post-translational modification preventing its use as a vaccine antigen [50]. fHbp has been demonstrated to be an effective protein antigen which can prevent serogroup B disease by eliciting serum bactericidal antibodies (SBAs), which activate the TP, leading to bacterial lysis, and are a widely accepted correlate of protection against IMD [51]. However, immune responses elicited by immunization with one fHbp are not necessarily protective against strains expressing another fHbp. For example, antibodies raised against B/v1 fHbps are not bactericidal against strains expressing A/v2 or v3 fHbp [1,2,52]. However, there is evidence of cross-reactive bactericidal activity within family A (i.e.*,* between v2 and 3 fHbps) which is not surprising given the relatedness of these proteins [1,2,53]. Consequently, Trumenba consists of two fHbps (belonging to families A and B) [54,55], while Bexsero has a different, single fHbp but with additional antigens [56].

Several attempts have been made to modify fHbp so that it elicits cross-protective SBAs. For example, regions of A/v2 and A/v3 fHbp have been introduced into a B/v1 fHbp. A total of 54 constructs were designed based on sequence diversity of fHbp and mAb binding sites, then empirically tested for their ability to elicit cross-variant fHbp SBA. One particular fHbp was the most promising at eliciting cross-variant responses [57] when fused in tandem to another meningococcal protein to enhance its stability.

**Figure 4:**
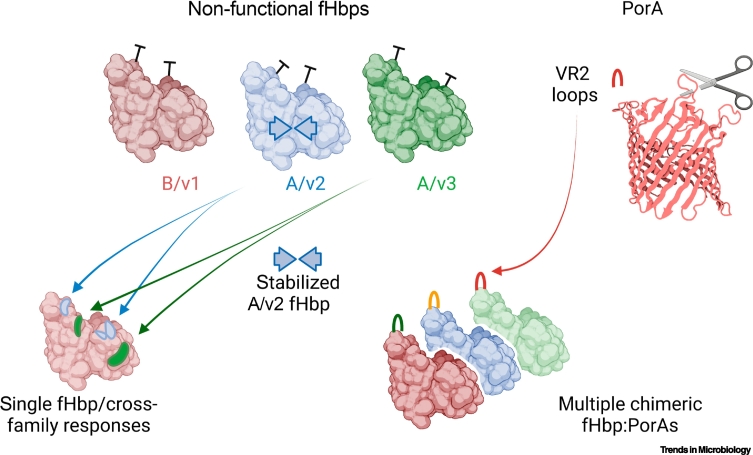
Next-generation factor H-binding protein (fHbp) vaccines. To induce cross-protective antibody responses, regions of A/v2 and A/v3 fHbps have been grafted onto a B/v1 fHbp; alternatively, multiple fHbps have been used (e.g., Trumenba). A/v2 fHbp is inherently unstable, and residues in the N-terminal barrel can be modified to increase its stability. Non-functional fHbps that are unable to bind to complement factor H (CFH) have been characterized as vaccine candidates. Structural vaccinology has been employed to exploit fHbp as a molecular scaffold to carry immunogenic surface loops of the integral outer-membrane protein PorA. These chimeric antigens can elicit bactericidal activity against both fHbp and PorA. Structures of A/v3 fHbp (PDB: 4AYI), and a *Neisseria* porin (PDB: 3VY8) are shown.

An alternative approach for generating broadly protective fHbp-based responses is to employ vaccines containing multiple fHbps, including B/v1, A/v2, and A/v3 proteins (Figure 4). Of note, no licensed vaccine contains a A/v2 fHbp, which has a relatively open N-terminal barrel with a melting temperature of below 40°C [7]. As vaccine antigens need to be stable and resistant to proteolysis, work has been performed to stabilize this fHbp. Introducing L130R and G133D substitutions in A/v2 fHbp increased the stability of its N-terminal barrel [58], although the effect of these changes on immunogenicity was not examined.

Clinical trials of fHbp-containing vaccines began before it was appreciated that the protein binds CFH with nanomolar affinity. As it is preferable that an immunogen does not tightly associate with a host protein [6,59], efforts have been made to develop fHbps which are non-functional and fail to bind CFH (Figure 4). The structure of fHbp:CFH paved the way for the development non-functional fHbps [6]. A single amino acid change in a B/v1 fHbp impeded CFH binding and retained immunogenicity [60], while comprehensive alanine scanning mutagenesis of B/v1, A/v2, and A/v3 fHbps at the CFH interface generated a catalog of substitutions that reduce fHbp:CFH affinity by more than an order of magnitude without affecting immunogenicity [7]. Error-prone PCR was also used to generate a library of *fhbp* mutants which were then expressed in *Escherichia coli* and tested for CFH binding [58]. Therefore a wide range of substitutions can be introduced into fHbp to make it non-functional. Non-functional fHbp has also been fused with cholera toxin B (CTB), with CTB acting as an adjuvant [61]. CTB-fusion proteins were more immunogenic than fHbp alone or mixtures of fHbp with CTB [61].

More recently, fHbp has been exploited as a molecular scaffold to display immunogenic epitopes from another meningococcal surface protein, PorA, generating chimeric fHbp:PorA vaccines (Figure 4). PorA is an integral outer-membrane protein (OMP) and the immunodominant antigen in meningococcal outer-membrane vesicle vaccines [62]. A major obstacle is producing PorA in its native conformation because of its hydrophobic, membrane-spanning domains. PorA has eight surface-exposed loops which are the target of SBA; most SBAs are directed at the fourth loop, called the variable region 2 (VR2) loop [63]. The soluble nature of the fHbp β-barrels was exploited to generate chimeric fHbp:PorA antigens with the PorA VR2 loop inserted into one of the β-barrels of fHbp [64]; structural analysis revealed that the PorA loops assume conformations in the chimeric antigens that are recognized by bactericidal mAbs. The chimeric antigen can elicit SBAs against both fHbp and PorA, providing a multivalent antigen in a single protein [64]. Furthermore, introduction of PorA loops in certain sites abrogates CFH binding. By combining both fHbp and PorA epitopes, vaccine coverage can be maximized by choosing the most prevalent fHbps and PorA loops.

Overall, various efforts have been made to enhance current *N. meningitidis* vaccines with advances in protein engineering and encompassing genomic epidemiology; improved vaccines covering a greater breadth of *N. meningitidis* strains are on the horizon.

## Concluding remarks

The interaction between fHbp and CFH provides a paradigm of how a single molecular interaction governs multiple aspects of host:pathogen relationships and can be exploited for translational benefits. As many pathogens recruit CFH to avoid immune detection, it is possible that defining the site of complement factor binding on other microbes could also be exploited for understanding virulence and vaccine design (see Outstanding questions). Indeed, it is possible that pathogens have evolved to recruit host factors that downregulate immune responses via their immunogenic surface molecules, subverting their recognition. The identification of microbial receptors for complement regulators might prove to be a productive approach for identifying vaccine candidates in other bacterial pathogens.

fHbp is an important virulence factor that is crucial for *N. meningitidis* pathogenesis. Levels of fHbp are carefully regulated on the surface of the bacterium in response to environmental triggers, allowing immune evasion during colonization and disease. Of note, temperature regulation of fHbp [23] would lead to low-level expression in the cooler niche of the nasopharynx, potentially reducing the impact of fHbp-based vaccines on carriage rates [65]. When the bacterium is exposed to higher temperatures and complement attack in the systemic circulation, the increase in fHbp levels would serve to protect the meningococcus from lysis. In this way, the high-affinity interaction of fHbp with CFH allows the bacterium to evade the most important aspect of host immunity against IMD, the complement system. This interaction has been pivotal in our understanding of the underlying mechanisms of strain virulence and host susceptibility in a remarkable convergence of findings from host and bacterial GWAS, with the most significant SNPs in human and meningococcal genomes mapping to either side of the fHbp:CFH interface. These findings could be used in the future to target vaccination to individuals at particular risk, through personalized immunization.

Importantly, knowledge of the atomic architecture of the fHbp:CFH interface and fHbp variation have paved the way for the application of structural vaccinology integrated with detailed knowledge of molecular epidemiology to rationally design stable, non-functional and chimeric fHbp antigens for next-generation vaccines. The presence of fHbp in commensal species such as *Neisseria cinerea* [66] means that the impact of fHbp-containing vaccines on the microbiome should be considered; fHbp-containing vaccines have the potential to perturb the nasopharyngeal flora, and affect the development of natural protective immunity. Despite this, fHbp-based vaccines are expected to play an important part in efforts to eliminate the threat of meningococcal disease across the world.

Outstanding questions Can the identification of sites of complement binding on other pathogens be exploited for vaccine design?

Does the meningococcus bind other CFHRs and what is the effect on complement evasion?

How do SNPs around CFH and CFHRs affect meningococcal susceptibility?

Do host polymorphisms contribute to the epidemic nature of disease seen in sub-Saharan Africa?

Will fHbp-based vaccines enhance protection offered by capsule polysaccharide conjugate vaccines?

Alt-text: Outstanding questions
